# Complete androgen insensitivity syndrome coexisting with müllerian duct remnants: a case report and literature review

**DOI:** 10.3389/fped.2024.1400319

**Published:** 2024-06-04

**Authors:** De-lu Chen, Song Guo, Qiu-li Chen, Shan-jiao Qiu, Yu-ying Xu, Jun Zhang, Hua-mei Ma, Yan-hong Li

**Affiliations:** Department of Pediatrics, The First Affiliated Hospital of Sun Yat-sen University, Guangzhou, China

**Keywords:** disorder of sexual development, testicular feminization syndrome, complete androgen insensitivity syndrome, Müllerian duct remnants, Müllerian duct regression

## Abstract

This study represents the first documentation of the coexistence of complete androgen insensitivity syndrome (CAIS) with Müllerian duct remnants (MDRs) in mainland China. Additionally, we provide a comprehensive review of the existing literature concerning CAIS with MDRs resulting from *androgen receptor (AR)* gene mutations. This study broadens the clinical spectrum of CAIS and offer novel insights for further exploration into Müllerian duct regression. A 14-year-old patient, initially raised as female, presented to the clinic with complaints of “primary amenorrhea.” Physical examination revealed the following: armpit hair (Tanner stage 2), breast development (Tanner stage 4 with bilateral breast nodule diameter of 7 cm), sparse pubic hair (Tanner stage 3), clitoris measuring 0.8 cm × 0.4 cm, separate urethral and vaginal openings, and absence of palpable masses in the bilateral groin or labia majora. The external genital virilization score was 0 points. Serum follicle-stimulating hormone level was 13.43 IU/L, serum luteinizing hormone level was 31.24 IU/L, and serum testosterone level was 14.95 nmol/L. Pelvic magnetic resonance imaging (MRI) did not reveal a uterus or bilateral fallopian tubes, but nodules on both sides of the pelvic wall indicated cryptorchidism. The karyotype was 46,XY. Genetic testing identified a maternal-derived hemizygous variation c.2359C > T (p.Arg787*) in the *AR* gene. During abdominal exploration, dysplastic testicles and a dysplastic uterus were discovered. Histopathological analysis revealed the presence of fallopian tube-like structures adjacent to the testicles. The CAIS patient documented in this study exhibited concurrent MDRs, thus expanding the spectrum of clinical manifestations of AIS. A review of prior literature suggests that the incidence of CAIS combined with histologically MDRs is not uncommon. Consequently, the identification of MDRs in AIS cases may represent an integral aspect of clinical diagnosis for this condition.

## 1 Introduction

Androgen insensitivity syndrome (AIS, OMIM #300068) is a rare X-linked recessive genetic disorder. AIS arises from pathogenic mutations in the androgen receptor (*AR*) gene, leading to complete or partial resistance to androgens, resulting in varying degrees of undervirilization. AIS represents a prevalent 46,XY disorder of sexual development (DSD) (1). In 1953, Morris initially described this syndrome as “testicular feminization syndrome" (2). There are no precise data regarding the prevalence of AIS. Research by Danish Berglund et al. suggested that the prevalence of AIS in 46,XY females was 6.4 per 100,000 live-born females (3). The estimated prevalence ranges from one in 20400 to one in 99,100 genetic males based on a proven molecular diagnosis (1). The *AR* gene is located at Xq11–12 and contains 8 coding exons, occupying more than 90 kb of genomic DNA (4). The *AR* protein, encoded by the *AR* gene, is part of the nuclear receptor superfamily and is also known as NR3C4 (nuclear receptor subfamily 3, Group C, Member 4) (5). The *AR* protein is composed of 920 amino acids and has a molecular weight of 110 kDa. This protein consists of four structural domains: the N-terminal transcription activation domain (NTD), the DNA-binding domain (DBD), the hinge region, and the ligand-binding domain (LBD) (6). The clinical manifestations and degree of androgen resistance can be classified into three types: complete AIS (CAIS), partial AIS (PAIS), and mild AIS (MAIS). In CAIS, patients exhibit pronounced feminization, characterized by female-appearing external genitalia and a blind-ended vaginal pouch. The testes may be located at any position along the descent pathway, including within the abdominal cavity, the inguinal canal, or the labia majora. The condition may be associated with either unilateral or bilateral inguinal hernias, and patients often present with primary amenorrhea after puberty. During embryonic development, the testes of AIS patients produce anti-Müllerian hormone (AMH), which induces regression of the Müllerian ducts (MDs), the anlage of the female internal reproductive organs (7). Therefore, the MD structure is often absent in AIS (1).

In this study, we report a case of CAIS coexisting with MD remnants (MDRs). Additionally, we provide a comprehensive summary of previously documented cases of CAIS, all confirmed through *AR* gene sequencing, which exhibited the presence of MDRs. This study was approved by the Medical Ethics Committee of the First Affiliated Hospital, Sun Yat-sen University [Approval No. (2023) 799].

## 2 Case description

### 2.1 General information

A 14-year-old Han Chinese girl raised as a female with nonconsanguineous parents was admitted to our clinic on August 1, 2023, with a chief complaint of breast development over the past four years without the onset of menstruation. The child exhibited good academic performance and female-like behavior. Breast development commenced four years prior, yet menarche had not occurred. Approximately one year ago (August 2022), a pelvic ultrasound performed at the primary hospital revealed an immature uterus and small gonads bilaterally. Karyotype analysis demonstrated a 46,XY genotype. For further diagnosis and treatment, the patient was admitted to our center on August 1, 2023. Since the onset of the condition, the patient has not manifested symptoms such as dizziness, headache, polydipsia, polyuria, vision impairment, hearing loss, or olfaction dysfunction.

#### 2.1.1 Past medical history

**Figure 1:**
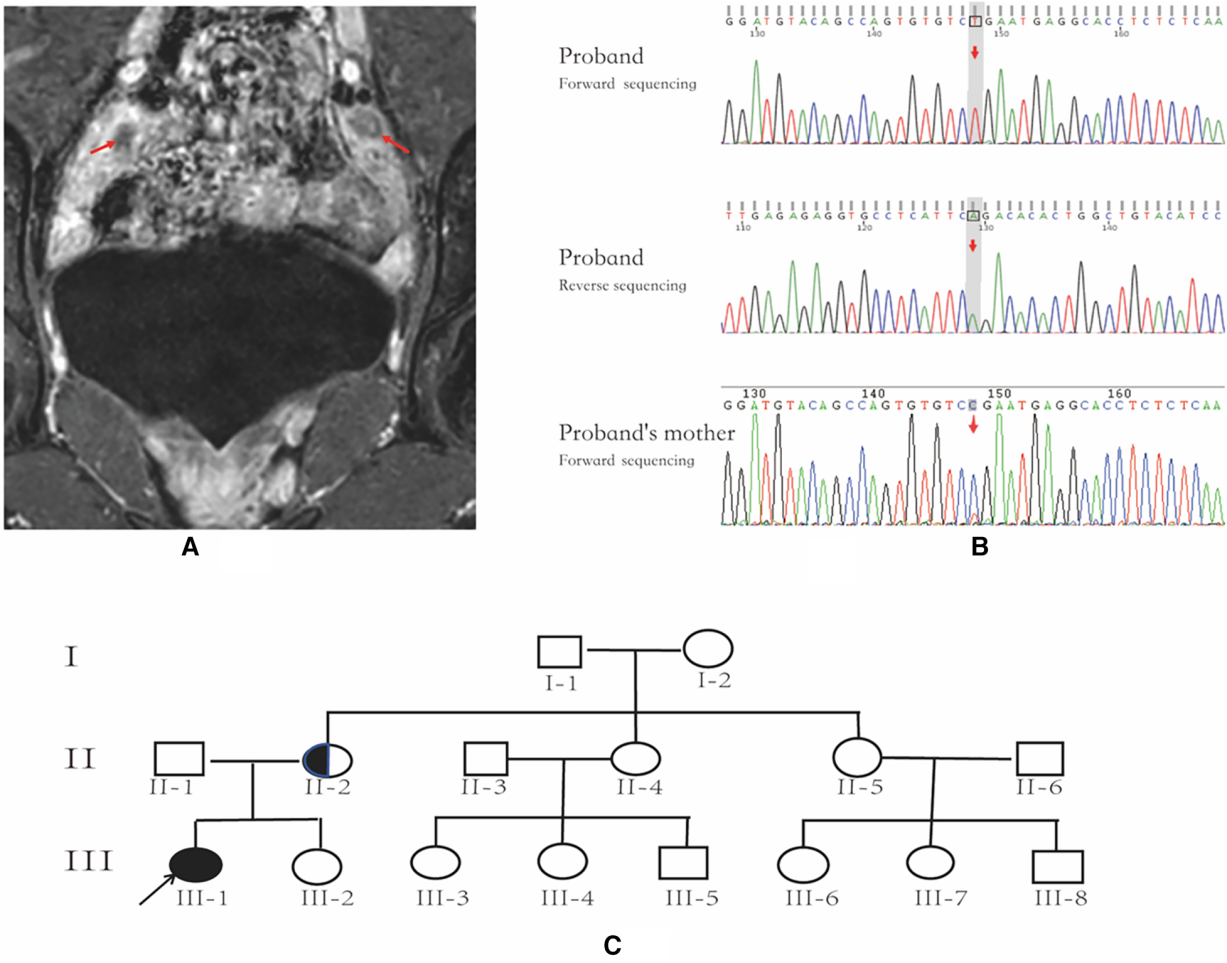
Pelvic MRI, *AR* gene sequencing chart and family pedigree. (**A**) Pelvic MR image; the arrows indicate bilateral testicles. (**B**) *AR* gene sequencing chart of the patient and the patient's mother; the arrow indicates the location of *AR* variant. The patient has an AR variant in hemizygosity (c.2359C > T, p.Arg787*), inherited from the mother. (**C**) family pedigree; the arrow indicates the proband, and the half-black/half-white circle is the carrier.

The patient was generally healthy with no history of exposure to toxins, radioactive substances, or contraceptives. Personal history: The patient was the first pregnancy and birth of the mother and was delivered at a gestational age of 39 weeks through an uneventful vaginal delivery. Her birth weight and length were 3.1 kg and 50 cm, respectively. The mother had an uneventful pregnancy course. Family history: The paternal and maternal heights were 165 cm and 155 cm, respectively. The target height was 153.5 ± 5 cm for the female, and 166.5 ± 5 cm for the male. Both parents had normal development during puberty. The patient's younger sister, aged 4 years and 10 months, exhibited normal growth and development. However, the parents declined to consent to a karyotype analysis for her. Her maternal grandmother and two maternal aunts (II-4, II-5; Figure 1B) had regular menstruation and normal fertility. Four maternal female cousins (III-3, III-4, III-6, III-7; Figure 1B) were unmarried and had never been pregnant but had regular menstruation. Two maternal male cousins (III-5 and III-8; Figure 1B) did not complain of micropenis.

#### 2.1.2 Physical examination

The patient's attire and mannerisms were feminine. Other physical measurements were recorded as follows: height 163 cm, weight 46 kg, and blood pressure 117/70 mmHg. The skin appeared smooth and devoid of acne. No palpable masses were identified in the inguinal region or labia majora. In terms of Tanner staging, the patient exhibited the following characteristics: Tanner stage 2 for axillary hair, Tanner stage 4 for breast development, with bilateral glandular nodules measuring 7 cm in diameter, and Tanner stage 3 for sparse pubic hair. The clitoris measured 0.8 cm × 0.4 cm, with separate urethral and vaginal openings. The external genitalia displayed a normal female appearance, and the External Masculinization Score (EMS) (8) was recorded as zero.

Further examination conducted at our center revealed that there were no abnormalities in the routine blood and urine tests. Additionally, there were no abnormalities detected in liver function, kidney function, blood glucose, blood lipids, or electrolytes. The 8AM cortisol concentration was 242.83 nmol/L, while the 8AM adrenocorticotropic hormone concentration was 3.77 pmol/L. Thyroid function was found to be within normal limits.

Serum levels of beta-human chorionic gonadotropin, alpha-fetoprotein, and carcinoembryonic antigen were within the normal range. FSH (13.43 IU/L), LH (31.24 IU/L), and T (14.95 nmol/L) were significantly elevated. Particularly noteworthy was the disproportionately higher serum LH level compared to the serum T level (Table 1). DHEAS (12.21 µmol/L), 17-*α*-OHP (34.55 nmol/L), and A4 (10.8 nmol/L) were also significantly elevated as summarized in Table 1. Her sex hormone-binding globulin (SHBG) concentration was 38.00 nmol/L, with a calculated free androgen index (FAI) of 39%. However, despite these findings, the patient did not exhibit clinical signs of hyperandrogenism, such as hirsutism or acne. Notably, the concentrations of anti-Müllerian hormone (AMH) and inhibin B (INHB) were markedly elevated at 206.51 ng/ml and 250.00 pg/ml, respectively, which are notably high for a female individual.

**Table 1:** Patient sex hormone levels before and after surgery.

Date	FSH (IU/l)	LH (IU/L)	E2 (pmol/L)	T (nmol/L)	P (nmol/L)	DHEAS (mmol/L)	A4 (nmol/L)	17-OHP (nmol/L)	AMH (ng/ml)	INHB (pg/ml)
2023-8-1	13.43	31.24	135.79	14.95	0.95	12.21	10.8	3.45	206.51	250.00
2023-8-21 (3 days after surgery)	71.1	40.95	<36.7	0.83	0.32	5.18	<1.05	0.67	87.86	20.00
Male RR (Tanner stage IV)	3.4–5.8	1.9–4.5	91.75–146.8	25.95–50.52	1.27–2.54	1.96–4.40	0.27–4.71	0.88–5.39	0.22–20.11	160.56–480.77
Female RR (Tanner stage IV)	4.6–8.0	3.4–13.4	136.71–314.88	2.42–5.88	1.27–3.82	1.71–4.07	0–6.16	0.52–7.00	1.04–17.93	10.00–466.16

RR: reference range; the reference ranges for E2, FSH, LH, T,P were obtained from Shenzhen, China (9). The reference ranges for the other hormones were obtained from unpublished data from our center.

The patient exhibited elevated levels of T, INHB, and AMH, indicating the likely presence of testicular tissue. A repeated pelvic ultrasound revealed nodules at the inguinal ring on both sides (approximately 1.2 cm × 0.6 cm on the left and 1.2 cm × 0.7 cm on the right), probably indicating the testes. However, the uterus and bilateral accessories were not visualized. Pelvic magnetic resonance imaging (MRI) showed no evidence of a uterus or bilateral accessories. The nodules observed on both sides of the pelvic wall were identified as testes (size 11 × 18 mm on the left side, size 24 × 11 mm on the right side), with a slightly high signal on T2-weighted imaging (T2WI) and marked enhancement (Figure 1A). Additionally, no abnormalities were detected on pituitary MRI.

Following the acquisition of informed consent from the patient and her parents, whole-exome genetic testing was conducted by Guangzhou Kingmed Center for Clinical Laboratory Co., Ltd. Molecular diagnosis unveiled an *AR* variant in hemizygosity (c.2359C > T, p.Arg787*), inherited from the mother (Figure 1B,C). Moreover, no aberrations were detected in the *AMH* and *AMHR2* genes. It is noteworthy that several researchers have previously documented the occurrence of the same site mutation in the *AR* gene among individuals diagnosed with AIS (10, 11).

### 2.2 Diagnosis and treatment

The patient was raised as a female and exhibited breast development and female external genitalia. Imaging of pelvic MRI suggested the presence of testicular tissue in the body. Elevated levels of T, AMH, and INHB, accompanied by increased FSH and LH levels, were noted. The karyotype was 46,XY. Based on these findings, a preliminary diagnosis of AIS with a completely female external genital phenotype was established. There is an increased risk of gonadal malignancies due to the assignment of a female gender to the patient and the likelihood of an CAIS diagnosis (genetic results were unavailable at the time of surgery).

**Figure 2:**
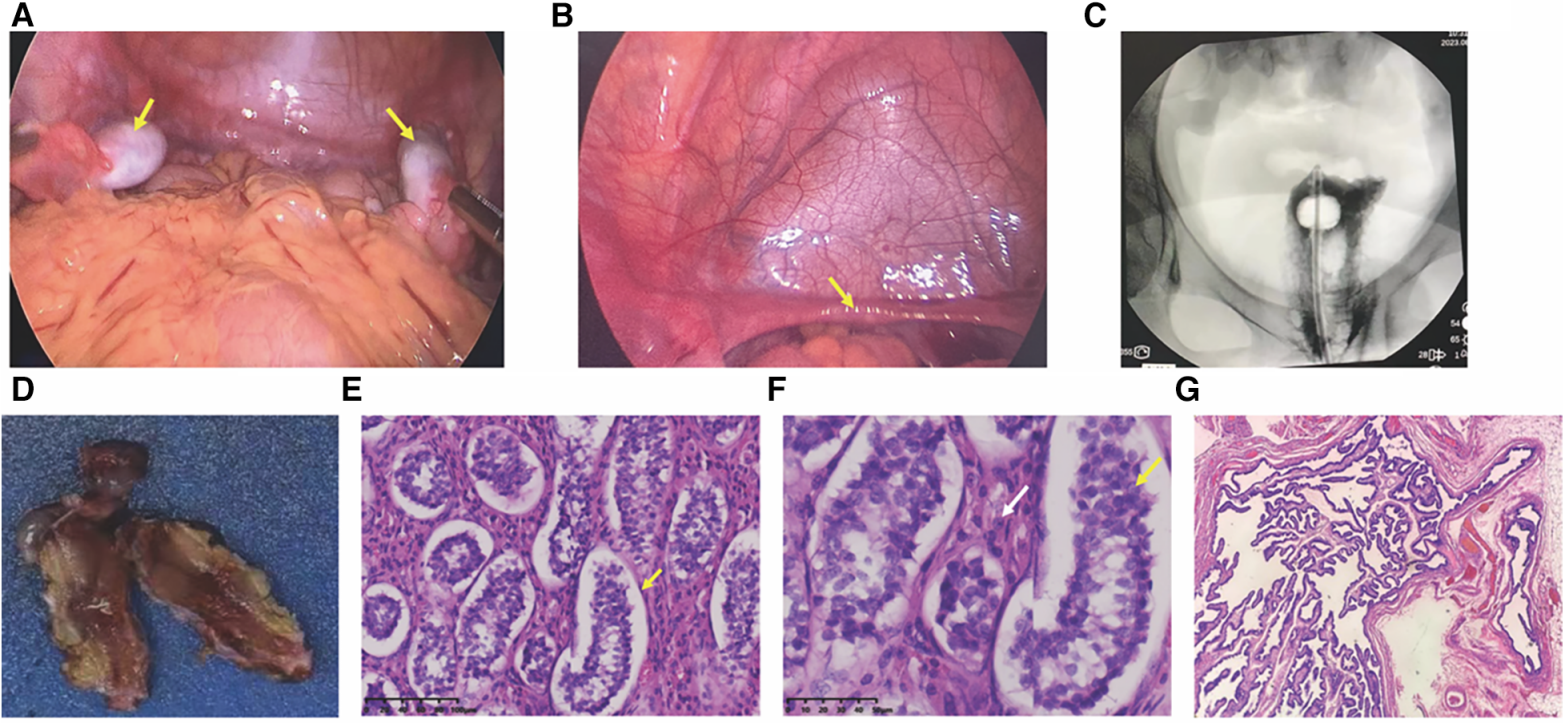
Laparoscopic view and histopathological pictures. (**A**) Laparoscopic view of bilateral gonads; arrows point to bilateral gonads. (**B**) Laparoscopic view of the hypoplastic uterus; arrows indicate a hypoplastic uterus. (**C**) Vaginogram; the patient has a vaginal length of approximately 8 cm, without the uterus. (**D**) Excised gonadal tissue; macroscopic aspect. (**E,F**) histopathological pictures. (**E**) histopathology revealing seminiferous tubules containing only Sertoli cells (×200); (**F**) Leydig cell hyperplasia indicated by white arrows (×400); (**G**) tubular structures resembling fallopian tubes (×40).

Following comprehensive communication with the parents and obtaining informed consent, the patient underwent laparoscopic examination. On August 18, 2023, in the pediatric surgery department, bilateral orchiectomy and vaginography procedures were conducted. Under laparoscopic view, the left internal ring was closed, while the right internal ring was still open. No vas deferens-like structures were identified at either internal ring. Gonad-like tissues approximately 2.5 cm × 1.0 cm in size were observed adjacent to the iliac vessels on both sides (Figure 2A). The bilateral gonads were connected to a uterine-like structure (Figure 2B). Subsequently, bilateral gonadal excisions were performed. Vaginography revealed a vaginal length of approximately 8 cm, with no discernible image of the uterus (Figure 2C).

Histopathology examination revealed bilateral gonadal tissue (Figure 2D) with immature seminiferous tubule-like structures under the microscope. The tubules contained only Sertoli cells (Figure 2E) with an absence of spermatogenic cells. Furthermore, significant proliferation of interstitial Leydig cells was observed (Figure 2F). Additionally, structures resembling fallopian tubes (Figure 2G) and mesenteric cysts were identified.

### 2.3 Follow-up

Three days post-surgery (August 21, 2023), repeated blood tests revealed the following results: E2 < 36.7 pmol/L, FSH 71.10 IU/L, LH 40.95 IU/L, T 0.83 nmol/L, P 0.32 nmol/L, DHEAS 5.18 µmol/L, A4 < 1.05 nmol/L, 17-OHP 0.67 nmol/L, AMH 87.86 ng/ml, and INHB 20.00 pg/ml (Table 1). Following orchiectomy, there was a notable decrease in E2, T, and androgen precursor hormones, accompanied by significant increases in FSH and LH levels. These findings are indicative of a hypergonadotropic hypogonadism state.

The patient was prescribed estrogen replacement therapy (0.5 mg of estradiol valerate once daily) to maintain secondary sexual characteristics such as breast development and to prevent osteoporosis. Additionally, the parents were counseled to disclose the patient's condition under suitable circumstances, with the provision of psychological support as needed. Furthermore, a plan to perform a hysterectomy for the underdeveloped uterus during the upcoming winter vacation was proposed.

### 2.4 Genetically confirmed CAIS patients coexisting with MDRs in the literature

The following are the inclusion criteria for CAIS patients in the literature: 1. Genetic confirmation of CAIS. 2. An MDR is present. 3. A comprehensive description of the Müllerian duct's structure and hormone profiles is provided. 4. Research that is available in full text.

Case search strategy: The search was conducted up to November 1, 2023. We searched for ([Androgen Insensitivity Syndrom*(MeSH Terms)] OR [Androgen Resistance Syndrom*(MeSH Terms)] OR [Male Pseudohermaphroditism Due to Androgen Insensitivity(MeSH Terms)] OR [Reifenstei* Syndrome(MeSH Terms)] OR [Testicular Feminizatio*(MeSH Terms)] OR [Androgen Receptor Deficien*(MeSH Terms)] OR [AR Deficien*(MeSH Terms)] OR [DHTR Deficien*(MeSH Terms)] OR [Dihydrotestosterone Receptor Deficien*(MeSH Terms)]) AND (Müllerian) in the PubMed.

There are 127 records according to the case search strategy. We manually screened patients diagnosed with CAIS coexisting with MDRs, resulting in a total of seventeen reports. After excluding six reports that did not undergo genetic testing (12–17) and two reports failed to obtain full text (18, 19), eleven cases remained for further analysis (20–28).

Data form eleven eligible CAIS patients were retrieved from the literature (20–28). Combined with the case reported in this article, a total of 12 patients of CAIS with MDRs have been documented (Table 2). Of these twelve patients, eight had uteruses (20–22, 24, 26, 28), including our patient. Among these individuals, patients 1 (20), 2 (21) and 12 underwent laparoscopy to find the uterus. In patients 4 (22), 11 (28), the uterus is found by MRI or ultrasound. In patients 6 (24), 8 (26), 9 (26), the uterus was confirmed pathologically. The initial clinical presentation, biochemical profile, and gonadal pathology of CAIS patients with MDRs were found to be similar to those without MDRs (1, 29). Patients 8 and 9 were from the same family, and shared identical genetic mutations (26). Among the cases reviewed, six patients were admitted for inguinal hernia, five for primary amenorrhea, and patient 11 for central precocious puberty with cyclical episodes of vaginal bloody spotting. All patients were diagnosed with CAIS confirmed by molecular testing and raised as females, with heights in the medium to upper average range. The age at diagnosis varied widely, spanning from birth to 22 years. Notably, patient 6 was diagnosed with CAIS *in utero*, and the pregnancy was terminated at 20 weeks of gestation (24). Among post-pubertal patients, varying degrees of breast development and sparse pubic hair growth were observed. Vaginal length ranged from 4 to 8 cm, indicating upper vaginal development from the MD in some patients. Specifically, six of the twelve patients had testes located in the inguinal canal, while five had testes in the abdominal cavity. Among the patients, one had a left testis in the abdominal cavity and another in the inguinal canal. According to the laparoscopic or pathological findings, fallopian structures were observed in 10 out of the 12 patients, uterine structures in 8 patients, and both fallopian and uterine structures in 6 patients. Notably, in patients older than 10 years, the concentration of LH was significantly elevated to >10 IU/L in 6 cases, with a mean value of 26.9 IU/L. Histological examination of the testes revealed Leydig cell hyperplasia in 4 out of 9 patients, while Sertoli cells were either normal or abundantly present. Germ cells were observed in younger patients, specifically patients 3 and 4 (22); In contrast, germ cells were nearly absent in older patients, specifically patients 8 and 9 (26), presenting as only Sertoli cells without germ cells. Genetic analysis identified mutations in the DNA-binding domain (DBD) of the *AR* gene in two out of the twelve patients, while eight patients (66.7%) in the ligand-binding domain (LBD). Among the twelve patients, one had a nonsense mutation in the *AR* gene, three had frameshift mutations, and eight (66.7%) had missense mutations.

**Table 2:** Characteristics of CAIS patients with MDRs reported in the literature.

Patient no.	**Age of diagnosis and chief complaint**	**Tanner stage**		**Vaginal length (cm)**	**Location of testes**	**MDR**	**Chemical data**				**histopathology**				***AR* gene**		
		**B^#^**	PH*				**FSH (IU/L)**	**LH (IU/L)**	**T (nmol/L)**	**AMH (ng/ml)**	**Leydig Cell**	**Sertoli cell**	**Germ cell**	**seminiferous tubule**	**Gene location/ amino acid transformation**	**Exon location/ functional domain**	**Variation of *AR* gene**
1 (20)	22-year-old, primary amenorrhea	3	5	8	Abdominal cavity	Uterus + bilateral fallopian	49.34	18.55	1.56	-	Not visible	Not visible	-	Not visible	c.1826_1830del/ (p.Arg609Lysfs*12)	3/DBD	Frame shift
2 (21)	9-year-old, inguinal hernia	1	1	Normal	Left abdominal cavity, right inguinal canal	Uterus + bilateral fallopian	6.9	0.6	4.77 (after hCG stimulation test)	-	-	-	-	Atrophied	c.2053G > A/ (p.Val685Ile)	4/LBD	Missense
3 (22)	10 months, bilateral inguinal hernia	1	1	-	Inguinal canal	Bilateral fallopian	-	-	-	-	-	Amount	Less	Normal	c.2608T>G/ (p.Met749Thr)	5/LBD	Missense
4 (22)	1-year-old and 1 month, bilateral inguinal hernia	1	1	-	Inguinal canal	Uterus + bilateral fallopian	-	-	-	-	Not visible	Amount	Some	Dense	c.2722T>A/ (p.Met787Lys)	6/LBD	Missense
5 (23)	14.5-year-old, primary amenorrhea	2-3	2	Short blind-ended vagina	Abdominal cavity	Right fallopian	3.7	18.3	18.68	-	Less	Existing	-	Diminished	c.T3004C/ (p.Leu881Pro)	8/LBD	Missense
6 (24)	Gestational age 20 weeks, -	-	-	-	Abdominal cavity	Uterus + fallopian	-	-	-	-	Hypertrophy and hyperplasia	Normal	Rare	Normal	c.2662A>T/ (p.Asp767Val)	5/LBD	Missense
7 (25)	14-year-old and 7 months, primary amenorrhea	3	3	-	Inguinal canal	Left fallopian	3.7	12.8	30.24	-	Existing	-	-	-	c.2111G > T/ (p.Ser704Ile)	4/LBD	Missense
8 (26)	12-year-old, inguinal hernia	5	2	7	Inguinal canal	Uterus	18	43.7	29.96	29.25	Hyperplasia	Amount	Not visible	Visible	c.1890delG/ (p.Lys631fs*2/)	4/?	Frame shift
9 (26)	18-year-old, primary amenorrhea, inguinal hernia	5	1	4	Abdominal cavity	Uterus + fallopian	11	37	8.37	24.25	Hyperplasia	Amount	Not visible	Dysplasia	c.1890delG/ (p.Lys631fs*2)	4/?	Frame shift
10 (27)	2 days, bilateral inguinal hernia	1	1	-	Inguinal canal	Left fallopian	<0.1	<0.1	1.45	-	Not visible	Normal	-	-	c.2255G > A/ (p.Trp752*)	5/LBD	Missense
11 (28)	6.8-year-old, central precocious puberty	2	1	-	Inguinal canal	Uterus	29.3	86.3	11.07	-	-	-	-	-	c.1752C > G/ (p.Phe584Leu)	2/DBD	Missense
12[Our study]	14-year-old, primary amenorrhea	4	3	8	Abdominal cavity	Uterus + fallopian	13.4	31.2	14.95	206.51	Hyperplasia	Existing	Not visible	Visible	c.2359C>T/ (p.Arg787*)	6/LBD	Nonsense

B#, breast, breast Tanner staging; PH*, pubic hair, pubic hair Tanner stage;?, According to the latest update on the https://androgendb.mcgill.ca/ website, it is not yet known in which region of the AR gene; DBD, DNA-binding domain; LBD, ligand-binding domain.

## 3 Discussion

AIS is a common 46,XY disorder of sexual development. In this study, we reported a case of CAIS attributed to a pathogenic variant in the *AR* gene coexisting with MDRs. Our findings underscore the importance of considering a diagnosis of CAIS even when MDRs are detected laparoscopically. Additionally, we observed that the initial clinical presentation, biochemical profiles, and gonadal pathology of CAIS patients with MDRs closely resemble those without MDRs. Previous reports indicate that the incidence of histologically MDRs in CAIS patients is approximately one third (30, 31). Consequently, the identification of MDRs should be incorporated into the clinical management strategies for CAIS.

This study reports a case of CAIS featuring a mutation at position c.2359 in the *AR* gene, resulting in a substitution of cytosine (C) with thymine (T). This alteration converts the arginine residue encoded by codon 787 of exon 6 into a termination codon, thereby prematurely terminating the synthesis of the *AR* protein. To our knowledge, there are two reported CAIS patients with the same *AR* gene mutation as our case by Dong et al. (10) and Kharrat et al. (11). Dong et al. (10) presented the presence of fallopian tubes in patients with CAIS(DSD 14 in Table 1 of the reference), while Kharrat et al. (11) reported a CAIS patient without MDRs (Patient 9 in Table 1 of the reference). This finding suggests that the presence of MDRs in CAIS patients may not be strongly related to genotype. Bermudez de la Vega et al. (28) reported that within the same family, CAIS caused by the same *AR* gene mutation can manifest as gender dysphoria when the patient is raised as a female or as central precocious puberty with periodic vaginal bleeding. This indicates that even the same genotype can have different clinical phenotypes, suggesting the role of environmental factors and epigenetics in the phenotype of CAIS.

This study provides a summary of the clinical data of genetically confirmed cases of CAIS with MDRs. Our findings indicate that the initial clinical presentation, biochemical profiles, and gonadal pathology of CAIS patients with MDRs closely resemble those without MDRs (1, 29).

A few of the patients included in Table 2 still require a great deal of discussion. The patient 1 (20) had a 46,XY karyotype and a complete female phenotype. As compared with serum LH (18.55 IU/L), the serum FSH level (49.34 IU/L) was substantially higher. In testicular histology, seminiferous tubules and sertoli-leydig cells were not present, and serum testosterone level was inadequate (0.45 ng/ml). These clinical signs do not align with the characteristics of classic AIS. Although we included this patient, testicular failure may be the more probable diagnosis. In patient 6 (24), under microscopy, not only MDRs but also Wolffian duct derivatives were discovered. It was hypothesized that elevated local concentrations of testosterone may trigger a reaction from mutant androgen receptors that still exhibited residual function, therefore promoting the development of WD. The AR gene variant of Patient 6 (24) is novel and the same mutation has not been reported previously. But the other variation at the codon 767 of AR gene (i.e., D767E) have been reported and verified to be CAIS in the Androgen Receptor Mutations Database (http://androgendb.mcgill.ca/). It is suggested that codon 767 is a crucial functional location inside the AR gene. Additionally, we used PolyPhen-2 to predict this variant of the AR gene, indicating that this mutation is most likely harmful.

Previous cohort studies have reported that approximately one third of CAIS patients present with coexisting histologically MDRs (30, 31), highlighting that the incidence of such coexisting MDRs in CAIS patients is not low. Although studies specifically addressing the risk of malignancy associated with MDRs in CAIS patients are lacking, existing literature on persistent Müllerian duct syndrome (PMDS) suggests a risk of MD malignancy ranging from 3%–8% (32, 33). Therefore, clinicians should exercise vigilance not only regarding the risk of gonadal malignancy in CAIS patients (34) but also regarding the presence of MDRs and their potential for malignancy.

It's indeed an intriguing phenomenon that MDRs are present alongside CAIS. Typically, in cases of CAIS, AMH released by the testes promotes the regression of MD around 8 weeks of gestation via a paracrine mechanism (35). As a result, Müllerian ducts regress in the majority of clinical instances of CAIS. However, while the testes have the capacity to synthesize AMH, it is plausible that insufficient or untimely AMH production occurred during the critical period of Müllerian duct regression. This possibility warrants further investigation.

Scholars suggested that the persistence of MDRs may be associated with deficiencies in the secretion or function of AMH (17). *AMH* and *AMHR2* gene testing was conducted on the patient as part of our investigation, yielding normal results. Picard JY et al. found that in 12% of patients with PMDS, there was no mutation detected in the *AMH* or *AMHR2* gene (36). This suggests that MD regression is governed by a complex gene regulatory network (GRN) (37). Currently, studies examining the role of the *AR* gene within this GRN are lacking. To understand why some AIS patients fail to experience the complete regression of MD, more research is necessary.

To the best of knowledge, this study represents the first report of the coexistence of CAIS with MDRs in mainland China. Additionally, we provide a comprehensive summary of the existing literature on CAIS with MDRs due to *AR* gene mutations. Our findings serve to broaden the clinical spectrum of CAIS and offer novel insights for further investigations into Müllerian duct regression. However, it is essential to acknowledge the limitation of this study, the current lack of a comprehensive explanation for the pathophysiology underlying the presence of MDRs in patients with CAIS, which warrants further research.

In conclusion, the presence of MDRs in children with CAIS expands the spectrum of clinical manifestations of CAIS. Moreover, this study provides an alternative perspective for fundamental research on MD regression, suggesting that the mechanism is not solely reliant on the *AMH–AMHR2* signaling pathway. Our research emphasizes the significance of integrating MDRs identification into the overall clinical management of CAIS, since the occurrence of histologically MDRs in CAIS patients is about one third.

## Data Availability

The original contributions presented in the study are included in the article/Supplementary Material, further inquiries can be directed to the corresponding authors.
